# Supplementary material for: Annihilation of exceptional points from different Dirac valleys in a 2D photonic system
Source: Nat Commun. 2022 Sep 12;13:5340. doi: 10.1038/s41467-022-33001-9 (PMC9468178; doi:10.1038/s41467-022-33001-9)
Supplement: Supplementary file 1 — Supplementary Notes [file 41467_2022_33001_MOESM1_ESM.pdf]

Annihilation of exceptional points from different Dirac valleys in a 2D photonic system. Supplementary Notes

M. Król, I. Septembre, P. Oliwa, M. Kędziora, K. Łempicka-Mirek, M. Muszyński, R. Mazur, P. Morawiak, W. Piecek, P. Kula, W. Bardyszewski, P. G. Lagoudakis, D. D. Solnyshkov, G. Malpuech, B. Piętka, and J. Szczytko

In these Supplementary Notes, we provide more details on the behavior of the Hermitian part of the Hamiltonian of our system. We demonstrate how the Hamiltonian parameters are extracted from the experiment, including the non-Hermiticity (linear dichroism). We present more details demonstrating the existence of exceptional points and their annihilation. Finally, we show that the winding of the real effective field constrains the possibility of a non-Hermitian topological transition with annihilation of the exceptional points: The annihilation is only possible if the total winding of the real effective field is zero.

I. TOPOLOGY OF THE HERMITIAN HAMILTONIAN: THEORY

In this section, we illustrate the topological transitions concerning the Hermitian part of the two-band Hamiltonian (1) of the main text describing the intersection of cavity modes with different numbers. This Hamiltonian is shown below for convenience, together with the definitions:

$$H_{\mathbf{k}}^{\mathrm{real}} = \begin{pmatrix} \frac{E_{\mathrm{H}}^{N+2}+E_{\mathrm{V}}^{N}}{2} + \frac{\hbar^2 k_x^2}{2m_x} + \frac{\hbar^2 k_y^2}{2m_y} & \Delta - \beta' k^2 - \beta(k_x - ik_y)^2 \\ \Delta - \beta' k^2 - \beta(k_x + ik_y)^2 & \frac{E_{\mathrm{H}}^{N+2}+E_{\mathrm{V}}^{N}}{2} + \frac{\hbar^2 k_x^2}{2m_x} + \frac{\hbar^2 k_y^2}{2m_y} \end{pmatrix}, \tag{S1}$$

where E_H^{N+2} and $m_H \sim N+2$ are the energy and mass, of the $N+2$th H-polarised mode and E_V^N and $m_V \sim N$, are the energy and mass of the V-polarised mode number N. k_x, k_y are the 2D wave vector components. The spin-independent masses m_x and m_y are determined by the birefringence (n_e, n_o) and the angle of the optical axis θ, according to the following expressions[1]:

$$\frac{1}{m_x} = \frac{1}{2m_0'}\left(\frac{n_{\mathrm{o}}^2\sin^2\theta + n_e^2\cos^2\theta + n_{\mathrm{e}}^2}{n_{\mathrm{e}}^2 n_{\mathrm{o}}^2}\right), \tag{S2}$$

$$\frac{1}{m_y} = \frac{1}{2m_0'}\left(\frac{n_{\mathrm{e}}^2 + n_{\mathrm{o}}^2}{n_{\mathrm{e}}^2 n_{\mathrm{o}}^2}\right), \tag{S3}$$

where m_0' is the mass at the central frequency (between the two modes in question). β is the magnitude of the TE-TM spin orbit coupling. $\beta' = \frac{\hbar^2(m_{\mathrm{V}}-m_{\mathrm{H}})}{2m_{\mathrm{H}}m_{\mathrm{V}}}$ and $\Delta = (E_{\mathrm{H}}^{N+2} - E_{\mathrm{V}}^{N})/2$. This Hermitian Hamiltonian can be written as a linear combination of identity and Pauli matrices which defines a real effective magnetic field Ω_{r} acting on the polarization pseudospin. The two non-zero components of the field are $\Omega_{\mathrm{r}}^x = \Delta - \beta' k^2 - \beta(k_x^2 - k_y^2)$ and $\Omega_{\mathrm{r}}^y = -2\beta k_x k_y$.

The pseudospin of a given eigenstate is either aligned or anti-aligned with the effective field (so far as the Hamiltonian remains Hermitian). Experimentally, this pseudospin corresponds to the Stokes vector of light, calculated using the following expressions:

$$\begin{aligned} S^1 &= \frac{I_{\mathrm{V}} - I_{\mathrm{H}}}{I_{\mathrm{V}} + I_{\mathrm{H}}}, \\ S^2 &= \frac{I_{\mathrm{D}} - I_{\mathrm{A}}}{I_{\mathrm{D}} + I_{\mathrm{A}}}, \\ S^3 &= \frac{I_{\mathrm{R}} - I_{\mathrm{L}}}{I_{\mathrm{R}} + I_{\mathrm{L}}}, \end{aligned} \tag{S4}$$

where the Is are the intensities in each of the polarizations, namely, V=Vertical, H=Horizontal, D=Diagonal, A=Anti-diagonal, R=Right-handed circularly polarized, L=Left-handed circularly polarized. These intensities can be measured experimentally or calculated theoretically as the absolute value squared of the eigenstate wavefunction in the corresponding basis. Each pseudospin component is an average value of the corresponding spin operator represented by a Pauli matrix.

The case $\beta' = 0$ has already been considered in[2,3]. The two parabolas are split at $k = 0$, with different possible signs of this splitting (band detuning).

For positive detuning, the TE-TM splitting compensates this splitting along k_x leading to a linear crossing. On the contrary, the TE-TM splitting is changing sign along k_y and adds to the $k = 0$ splitting. As a result, along k_y the two parabola get away the one with respect to the other and do not cross. The resulting spectrum shows two tilted Dirac cones at $\pm k_{0x}$. The winding number of the effective field around these two points is the

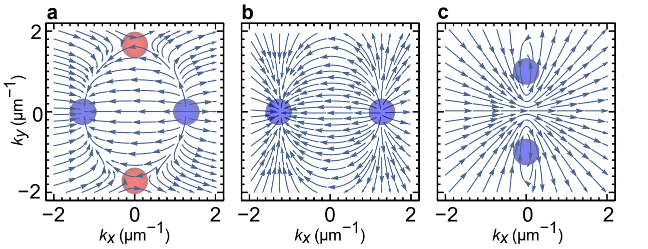

FIG. S1: **Pseudospin textures for the Hermitian Hamiltonian**: **a** trivial case $\beta < \beta'$, **b,c** topologically non-trivial case $\beta > \beta'$ with positive and negative detunings. The red marks shows the Dirac points of winding number $+1$ and the blue marks the Dirac points of winding number -1.

same $(+1)$, as shown in Fig. S1b showing the distribution of the pseudospin (and effective field) texture in the reciprocal space. This winding is inherited from the winding number 2 of the TE and TM modes. The Berry topological charge is $+1/2$. This topological phase remains while β' increases up to $\beta' = \beta$ where two new crossing points between the parabola appear at k_{0y} which sets at infinity when the transition occurs. These crossing points correspond to tilted Dirac cones. The winding number of the in-plane pseudospin around these two points is (-1) and the Berry topological charge (-1/2), as shown on Figure S1b. The sum of the topological charges of the 4 Dirac points is zero and the bands are overall topologically trivial which allows the non-Hermitian topological transition we explore in the main text.

For negative detuning (Fig. S1c), the winding of the two Dirac points does not change, but their positions in reciprocal space are rotated by $\pi/2$ and the particular field distribution changes from monopolar to Rashba.

II. TOPOLOGY OF THE HERMITIAN HAMILTONIAN: EXPERIMENT

Fig. S2 presents realization of the case with winding 0, discussed in detail in the main text. Fig. S2a,b presents experimental dispersion relation along k_x and k_y directions observed with 1.39 V applied to ITO electrodes. At this voltage, the numbers of the modes differ by 2, and both modes cross each other along both wave vector directions. Experimentally determined pseudospin texture in S_1–S_2 plane of the lower energy band is plotted in Fig. S2c. It matches the Berreman matrix model presented in Fig. S2d, showing 0 total winding.

With amplitude of external voltage of 11 V modes with the same mode numbers are close to degenerate, as shown on experimental dispersion relations in Fig. S3. In this case modes crosses each other only along k_y direction. Resulting pseudospin texture in S_1–S_2 plane shows two 2D monopoles with total winding number of 2 in both experiment Fig. S3c and Berreman model simulations Fig. S3d.

The Dirac points appear along the Y axis because of

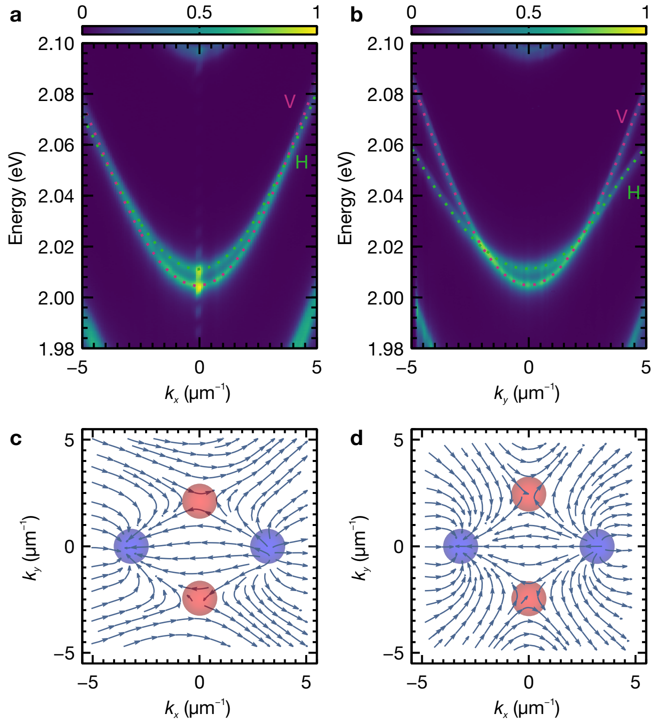

FIG. S2: **4 diabolical points case**. Experimental dispersion relation along **a** k_x and **b** k_y directions observed at 1.39 V applied to the microcavity, corresponding to the case with total winding 0. Energy of the bands visible in horizontal (vertical) polarization is marked by green (purple) dashed line. **c** Experimental and **d** simulated by Berreman method pseudospin texture in S_1–S_2 plane of lower energy band.

the negative value of the detuning in the experiment, but their winding is that of the TE-TM field, as expected. These epxeriments were performed on the sample 2, in a region with an LC layer thickness of around 3.9 μm. Panels (c) of Fig. S2 and Fig. S3, demonstrating the experimentally extracted pseudospin texture exhibiting different total winding, are shown in Fig. 3 of the main text.

III. HAMILTONIAN PARAMETERS

The parameters of the Hermitian Hamiltonian (1) are extracted from the fit of the dispersion in the two perpendicular directions. The fits are shown in Fig. S4. In a given direction, the average of the two parabolicity coefficients (inverse masses) of the two polarizations, provides the value of $1/m_x$ and $1/m_y$. Then, the difference between the two parabolicites in the two polarizations is $\beta + \beta'$ for Y direction and $\beta - \beta'$ for X direction. Fitting both allows to extract β and β' separately.

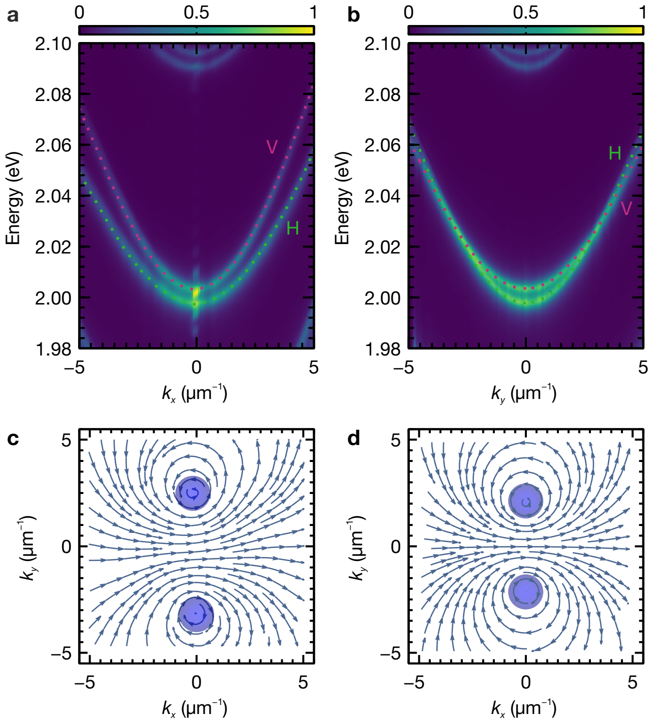

FIG. S3: **2 diabolical points case**. Experimental dispersion relation along **a** k_x and **b** k_y directions, observed at 11 V applied to the microcavity, corresponding to the case with winding 2. Energy of the bands visible in horizontal (vertical) polarization is marked by green (purple) dashed line. **c** Experimental and **d** simulated by Berreman method pseudospin texture in S_1–S_2 plane of lower energy band.

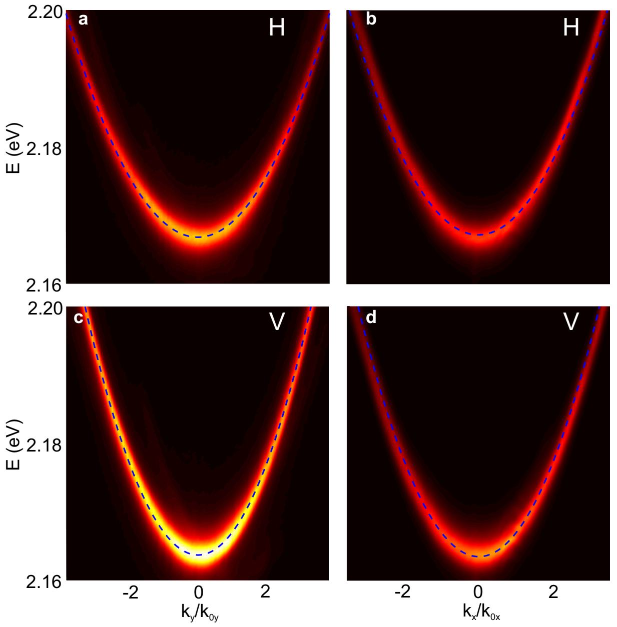

FIG. S4: **Fit of the dispersions.** Fit of the polarization-resolved dispersions in the two perpendicular directions. The false color map shows the transmission as a function of wave vector and energy. Fitting allows to obtain the spin-independent masses m_x and m_y, as well as the polarization splittings β and β'.

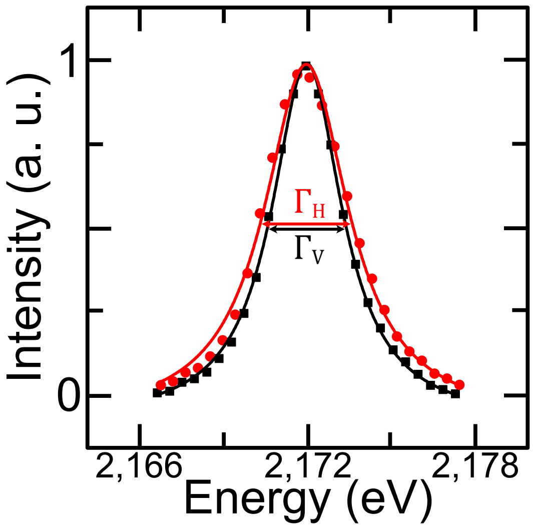

FIG. S5: **Polarization-resolved transmission spectrum.** The transmission in polarizations H and V, measured at the position of the diabolical point and plotted as a function of energy. The difference of linewidths is approximately 13%.

IV. NON-HERMITIAN PARAMETER

In Fig. S5, we show an example of a spectrum of transmission in two polarizations, clearly demonstrating different linewidths. The experimental points for each of the two polarizations are fitted with the Voigt function, as explained in Methods. We stress that while the non-Hermiticity of the Hamiltonian is a single constant, the linewidth at each particular wave vector is determined by the imaginary part of the corresponding eigenstate. Only at the positions of the Dirac points of the Hermitian Hamiltonian does the linewidth of the eigenstates correspond directly to the non-Hermiticity of the Hamiltonian $\delta\Gamma$. It is also the maximal possible imaginary part of the energy that could be observed for any wave vector. This is what we use to extract the non-Hermitian parameter $\delta\Gamma$.

V. EXCEPTIONAL POINTS AND THEIR ANNIHILATION

In this section, we present more information on exceptional points.

The 2D images shown in Fig. 4 of the main text do not allow to indicate the uncertainties, which are important to prove that the exceptional points are indeed present in our system and that the transition associated with the annihilation of the EPs really takes place.

In Fig. S6a,b we plot the real and imaginary parts (respectively) of the complex energies extracted along the

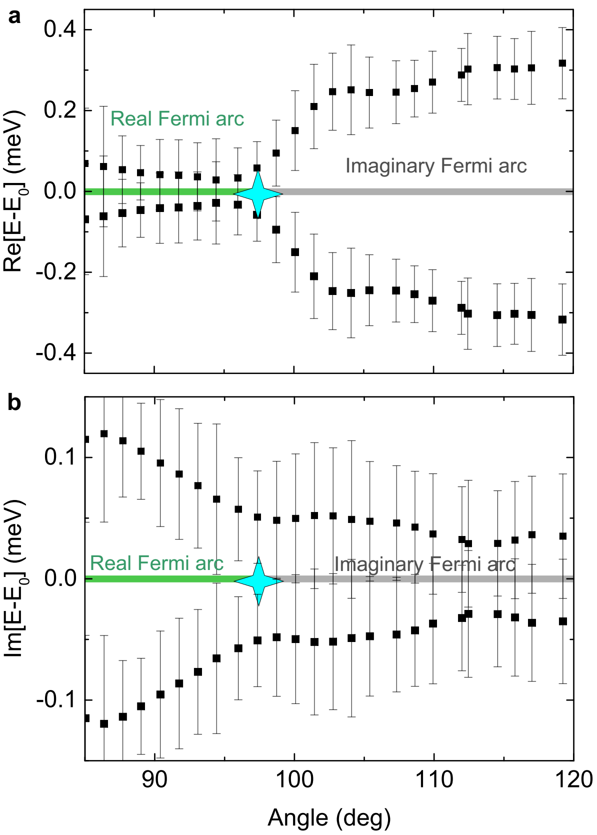

FIG. S6: **Exceptional point. a** Real and **b** imaginary parts of extracted energies as a function of angle along the Fermi arc in vicinity of an exceptional point.

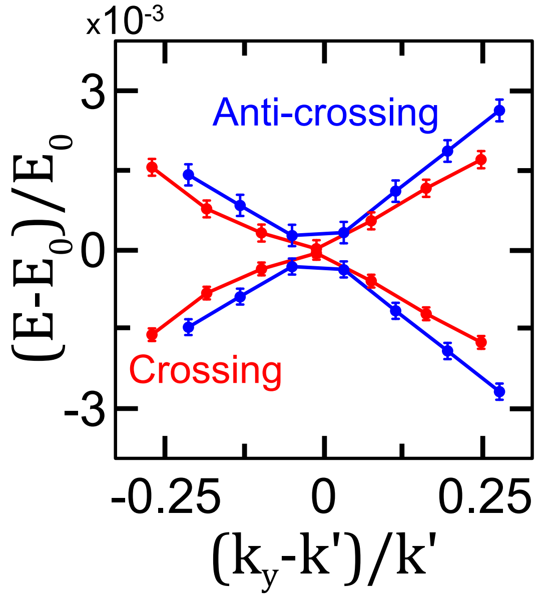

FIG. S7: **Crossing and anti-crossing of the bands.** Real part of the energies near the top-right corner of the Fermi arc. Before the annihilation of EPs, an imaginary Fermi arc still exists, which is shown here by the anti-crossing of the real energies. On the contrary, when the EPs are annihilated, the real Fermi arc forms a closed loop and the imaginary Fermi arc disappears. Thus, the bands cross.

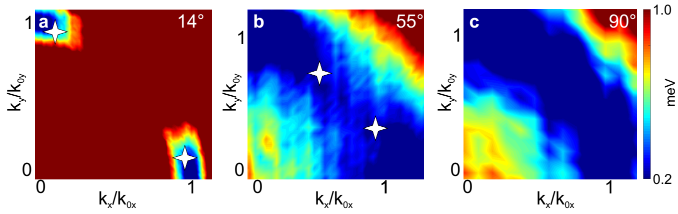

FIG. S8: **Exceptional point motion and annihilation.** Sample 2. Difference between the real energies $|\Delta E|$ for 3 particular detunings: **a** $2\Delta = 3.4$ meV, $\chi = 0.175$, real Fermi arc 14°; **b** $2\Delta = 0.7$ meV, $\chi = 0.85$, real Fermi arc 55°; **c** $2\Delta = 0.4$ meV, $\chi = 1.48$, real Fermi arc 90°.

Fermi arc in vicinity of a particular exceptional point located in the top part of Fig. 4 of the main text, with the error bars indicating the uncertainty of the extraction. Both real and imaginary Fermi arcs are clearly visible. The exceptional point is marked with a star. The degeneracy of the real part along the real Fermi arc and that of the imaginary part along the imaginary Fermi arc are confirmed by the overlap of the error bars.

We also plot in Fig. S7 the real part of the energies in the crossing (EPs annihilated) and anti-crossing (EPs present) case. This sums up as a cross-section of Fig. 4a,d of the main text near the top right corner of the Fermi arc denoted by k'. E_0 is the difference between the real parts of the upper and lower branches, and is in general different for the two cases. In one case, this cross-section crosses an imaginary Fermi arc, and in the other case, a real Fermi arc that forms a full circle.

Most of the results shown in the main text and all results presented above in the Supplemental Notes were obtained with Sample 1. To demonstrate that the observed behavior is universal, and does not depend on a particular sample, we have performed extra measurements with Sample 2, characterized by different parameters. Figure 4m of the main text presents the length of the real Fermi arc as a function of non-Hermiticity χ for both samples. In Fig. S8, we present three examples of reciprocal space images of the difference in the real part of the extracted energies $|\Delta E|$, corresponding to 3 different detunings Δ and values of non-Hermiticity χ (see caption). The exceptional points are shown with white stars. The angle marked in the top right corner of each panel indicates the length of the real Fermi arc, which can be obtained from the figure by subtracting the imaginary Fermi arc (between the stars) from 90 degrees.

The relevant parameters of the sample 2 are given below: $\beta = 0.23 \pm 0.01$ meVμm^2, $\beta' = 0.35 \pm 0.01$ meVμm^2, $2\delta\Gamma = 0.5 \pm 0.07$ meV.

VI. HERMITIAN WINDING AND ANNIHILATION OF EXCEPTIONAL POINTS

We consider a two-band system described by a Hamiltonian depending on a two-dimensional wave vector. The Hermitian part of the Hamiltonian is characterized by a presence of diabolical (Dirac) points. The two-band nature allows writing the Hamiltonian as a superposition of Pauli matrices. For many typical situations, such Hamiltonians can be written using only two Pauli matrices that we can choose to be σ_x and σ_y (massless Dirac Hamiltonian, Rashba and Dresselhaus spin-orbit couplings, TE-TM spin-orbit coupling for photons), whereas the third Pauli matrix σ_z, responsible for a symmetry breaking, opens the gap at the diabolical points (the mass term in the Dirac Hamiltonian or the Zeeman splitting for electrons and photonic modes). In this case, it is possible to characterize each of the diabolical points and the whole reciprocal space by winding numbers, which, in turn, determine the topology (the Chern number) of the bands once the gap is opened.

We then consider the evolution of the Hamiltonian with an addition of non-Hermiticity, described by a single constant parameter (independent of the wave vector). Without loss of generality, we consider the non-Hermitian contribution to be described by the Pauli matrix σ_x. In presence of the non-Hermiticity, each of the diabolical points gives rise to two exceptional points.

Thanks to the use of the Pauli matrices, it is possible to map the system's Hamiltonian to a spin-1/2 in a magnetic field, using the so-called pseudospin formalism, where the terms of the Hamiltonian are considered as an effective field. In the presence of a non-Hermiticity, this effective field has both real and imaginary components, $\mathbf{\Omega} = \mathbf{\Omega}' + i\mathbf{\Omega}''$. An exceptional point requires the real and imaginary parts of the effective field to be equal to each other in magnitude and perpendicular in direction: $\Omega' = \Omega''$ and $\mathbf{\Omega}' \perp \mathbf{\Omega}''$.

Since in our case the direction of the imaginary field is fixed $\mathbf{\Omega}'' = \Omega''\boldsymbol{e}_x$, and the real field is in the XY plane, the exceptional points can only be found where the x-component of the real field is zero $\mathbf{\Omega}' = \Omega'\boldsymbol{e}_y \Leftrightarrow \Omega'_x = 0$. This condition constrains their evolution in the reciprocal space when the imaginary field is increased $\Omega'' \to \infty$ (or the real field is decreased $\Omega' \to 0$). The location of the ensemble of the points $\Omega'_x = 0$ is determined by the winding of the real field at the diabolical points. With the increase of Ω'', the exceptional points are moving towards infinity. **If no points, where the condition for the real part $\Omega'_x = 0$ is satisfied, can be found at infinity, then the exceptional points must annihilate for some finite critical value Ω''_c.** Two cases are possible:

1. The overall winding of the real field is a non-zero integer. In this case, the orientation angle θ_r of the real field at infinity changes at least once between $\theta_\mathrm{r} = 0$ and $\theta_\mathrm{r} = 2\pi$, necessarily going through $\pi/2$ and $3\pi/2$, where $\Omega'_x = 0$. Therefore, the exceptional points can be found for any value of Ω'' up to infinity, and thus they cannot annihilate (or if they annihilate accidentally, they must then reappear when Ω'' is increased).

2. The overall winding of the real field is zero. In this case, the orientation angle of the real field θ_r at infinity is constrained to an interval which may not include $\pi/2$ or $3\pi/2$. In this case, the exceptional points cannot be present at infinity, and therefore they must annihilate for a finite value of Ω''_c.

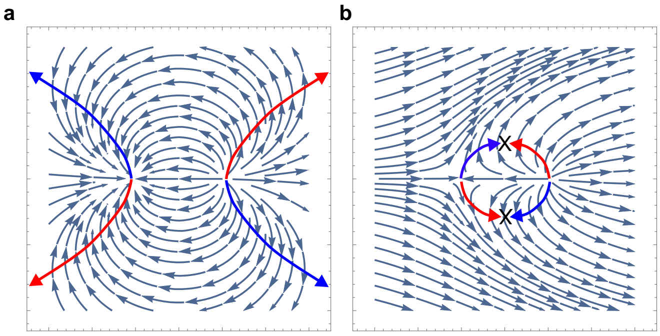

FIG. S9: **Hermitian winding and annihilation of exceptional points. a** Non-zero total winding of the real field, the exceptional points do not annihilate. **b** Zero total winding of the real field, annihilation of exceptional points. The trajectories of the exceptional points are shown with red and blue arrows (corresponding to the winding of the exceptional points themselves). Crosses mark the annihilation points.

The two situations are illustrated in Fig. S9. Panel a shows the case with total winding 2 with a dipolar texture typical for the TE-TM field. The trajectories of the exceptional points are shown with red and blue lines, which are defined by the condition $\Omega'_x = 0$ (vertical orientation of the real field). At a large scale, the relative position of the two diabolical points does not play any role and the texture is given by $\theta_r = 2\varphi$ (where φ is the polar angle of the wave vector). The exceptional points at infinity are therefore necessarily located at $\varphi = \pm\pi/4, \pm 3\pi/4$ (while initially they move from the diabolical points in the vertical direction).

The second situation is illustrated by Fig. S9b, where the overall winding of the real field is zero. The angle of the real field at infinity is $\theta_\mathrm{r} = 0$ and there can be no exceptional points. The condition $\Omega'_x = 0$ (vertical real field) gives the trajectories of the exceptional points shown in the figure, with the annihilation points marked with crosses.

Finally, the winding of the exceptional points themselves is shown with a color of the arrows (red and blue).

The winding of an exceptional point is the winding of the phase of the complex eigenenergies. Indeed, the energy of a second-order exceptional point is given by

$$E = \pm\sqrt{\alpha q} e^{iw\phi/2} \qquad \text{(S5)}$$

where $w = \pm 1$ is precisely the winding number and q is the absolute value of the parameter controlling the deviation from the exceptional point, for example, the wave vector (measured from the exceptional point). This eigenenergy can be obtained as a solution for different Hamiltonians, with different variation of real and imaginary effective fields. Below, we show that when the imaginary effective field $\mathbf{\Omega}''$ is constant, the winding of the exceptional point can be easily determined from the texture of the real effective field $\mathbf{\Omega}'$. The most general form of a Hamiltonian of such type is

$$\begin{aligned}\hat{H} &= \alpha q \left(\sigma_x \cos\varphi + iw\sigma_y \sin\varphi\right) \\ &+ a((\sigma_x \cos\theta + \sigma_y \sin\theta) \\ &+ ia\left(\sigma_x \cos(\theta \pm \pi/2) + \sigma_y \sin(\theta \pm \pi/2)\right)\end{aligned} \tag{S6}$$

where the a gives the values of the real and imaginary effective field oriented at an angle θ and $\theta + \pi/2$ respectively, equal and perpendicular at the exceptional point, w is the winding of the parameter-dependent part of the real effective field and α is its strength. The eigenenergies of this Hamiltonian are given by

$$E \approx \pm\sqrt{q}\sqrt{2\alpha a}e^{\pm iw\varphi/2}e^{-i\theta/2} \tag{S7}$$

We see that the winding of an exceptional point is determined by the winding w of the original diabolical point, modified by the relative angle of the real and imaginary fields at each exceptional point $\pm\pi/2$.

Supplementary References

[1] K. Rechcińska, M. Król, R. Mazur, P. Morawiak, R. Mirek, K. Łempicka, W. Bardyszewski, M. Matuszewski, P. Kula, W. Piecek, et al., Science **366**, 727 (2019).

[2] H. Terças, H. Flayac, D. D. Solnyshkov, and G. Malpuech, Phys. Rev. Lett. **112**, 066402 (2014), URL `https://link.aps.org/doi/10.1103/PhysRevLett.112.066402`.

[3] A. Gianfrate, O. Bleu, L. Dominici, V. Ardizzone, M. De Giorgi, D. Ballarini, G. Lerario, K. West, L. Pfeiffer, D. Solnyshkov, et al., Nature **578**, 381 (2020).
